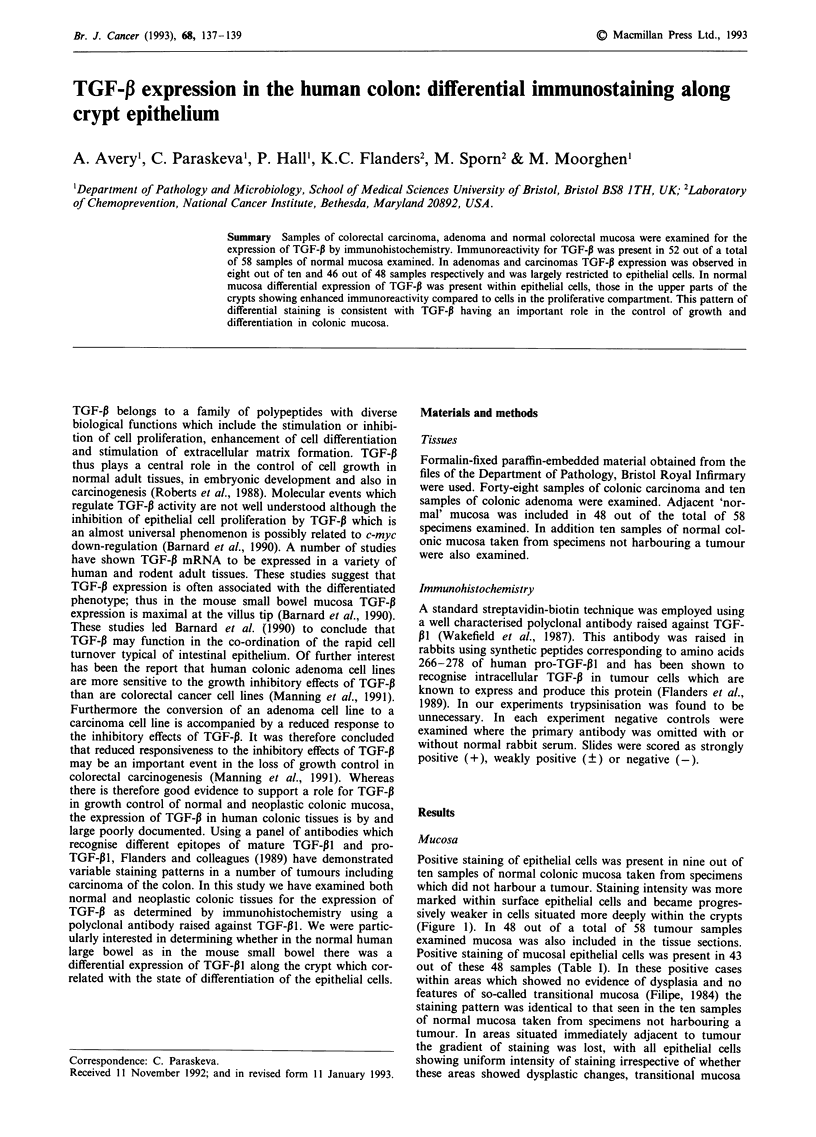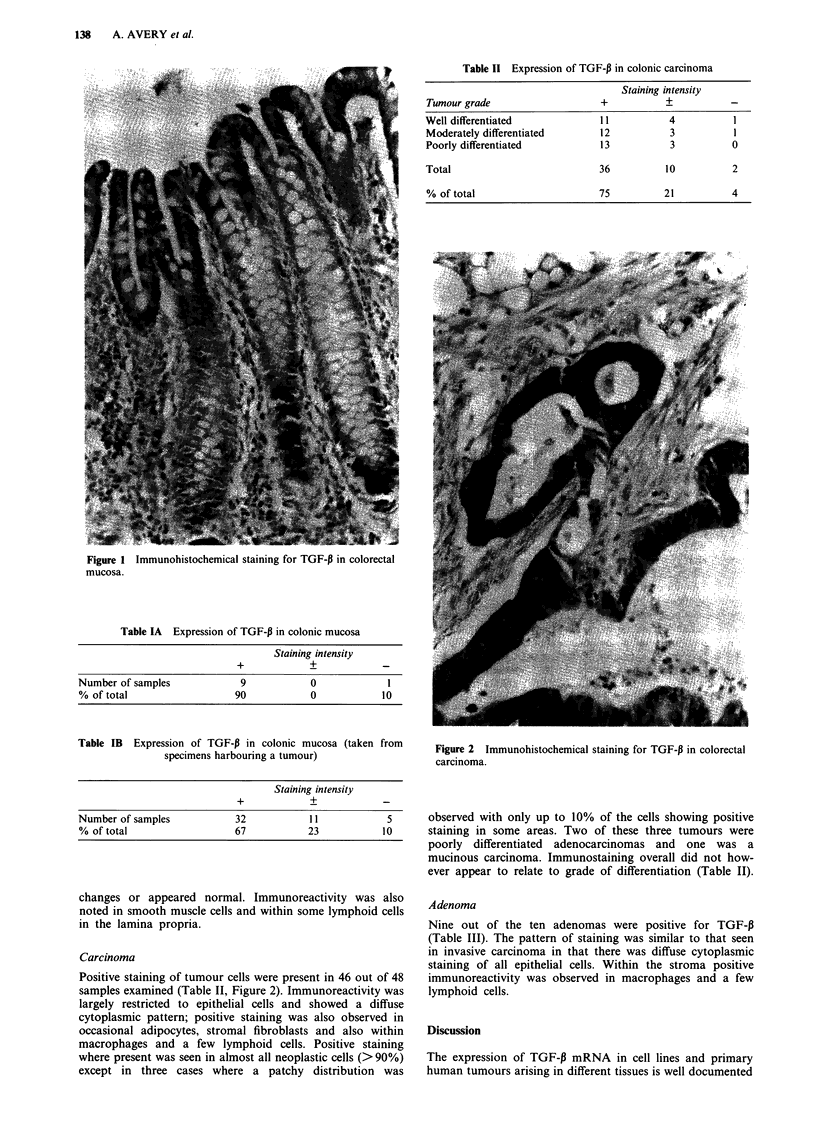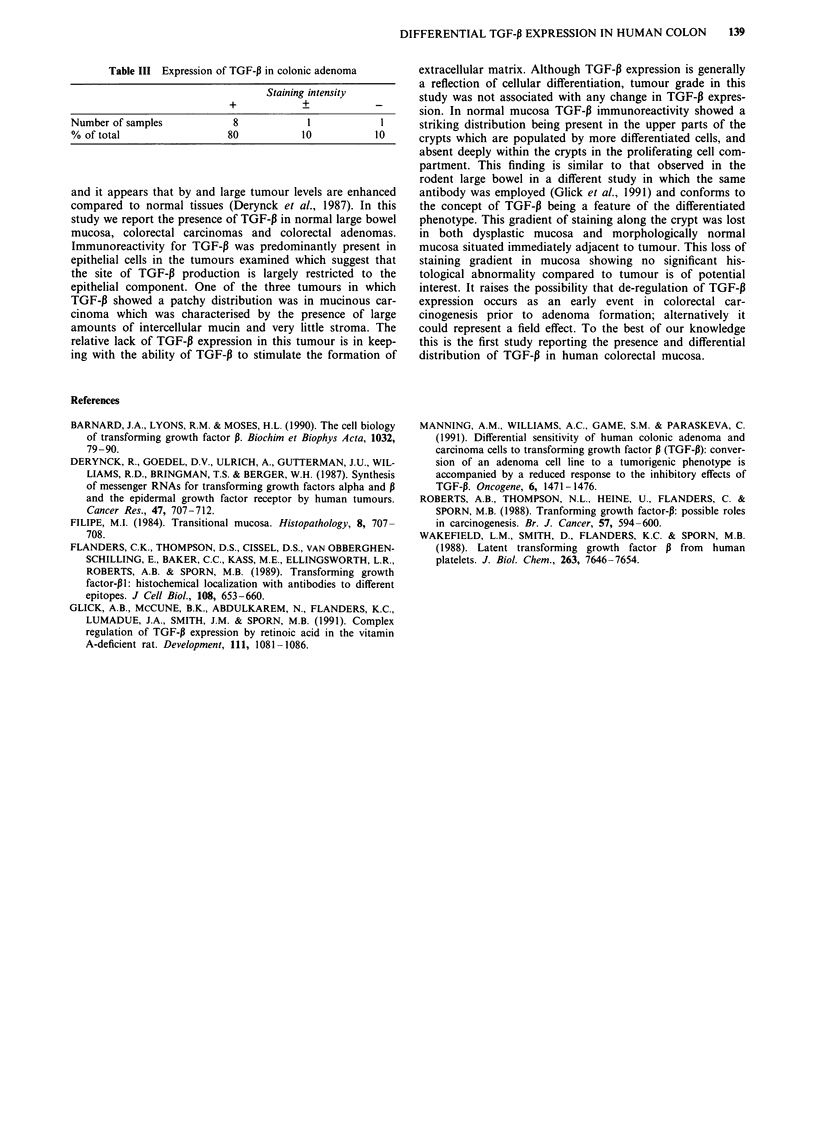# TGF-beta expression in the human colon: differential immunostaining along crypt epithelium.

**DOI:** 10.1038/bjc.1993.301

**Published:** 1993-07

**Authors:** A. Avery, C. Paraskeva, P. Hall, K. C. Flanders, M. Sporn, M. Moorghen

**Affiliations:** Department of Pathology and Microbiology, School of Medical Sciences University of Bristol, UK.

## Abstract

**Images:**


Br. J. Cancer (1993), 68, 137–139 © Macmillan Press Ltd., 1993

### TGF-β expression in the human colon: differential immunostaining along crypt epithelium

A. Avery[1], C. Paraskeva[1], P. Hall[1], K.C. Flanders[2], M. Sporn[2] & M. Moorghen[1]

[1]*Department of Pathology and Microbiology, School of Medical Sciences University of Bristol, Bristol BS8 1TH, UK;* [2]*Laboratory of Chemoprevention, National Cancer Institute, Bethesda, Maryland 20892, USA.*

**Summary** Samples of colorectal carcinoma, adenoma and normal colorectal mucosa were examined for the expression of TGF-β by immunohistochemistry. Immunoreactivity for TGF-β was present in 52 out of a total of 58 samples of normal mucosa examined. In adenomas and carcinomas TGF-β expression was observed in eight out of ten and 46 out of 48 samples respectively and was largely restricted to epithelial cells. In normal mucosa differential expression of TGF-β was present within epithelial cells, those in the upper parts of the crypts showing enhanced immunoreactivity compared to cells in the proliferative compartment. This pattern of differential staining is consistent with TGF-β having an important role in the control of growth and differentiation in colonic mucosa.

TGF-β belongs to a family of polypeptides with diverse biological functions which include the stimulation or inhibition of cell proliferation, enhancement of cell differentiation and stimulation of extracellular matrix formation. TGF-β thus plays a central role in the control of cell growth in normal adult tissues, in embryonic development and also in carcinogenesis (Roberts *et al.*, 1988). Molecular events which regulate TGF-β activity are not well understood although the inhibition of epithelial cell proliferation by TGF-β which is an almost universal phenomenon is possibly related to *c-myc* down-regulation (Barnard *et al.*, 1990). A number of studies have shown TGF-β mRNA to be expressed in a variety of human and rodent adult tissues. These studies suggest that TGF-β expression is often associated with the differentiated phenotype; thus in the mouse small bowel mucosa TGF-β expression is maximal at the villus tip (Barnard *et al.*, 1990). These studies led Barnard *et al.* (1990) to conclude that TGF-β may function in the co-ordination of the rapid cell turnover typical of intestinal epithelium. Of further interest has been the report that human colonic adenoma cell lines are more sensitive to the growth inhibitory effects of TGF-β than are colorectal cancer cell lines (Manning *et al.*, 1991). Furthermore the conversion of an adenoma cell line to a carcinoma cell line is accompanied by a reduced response to the inhibitory effects of TGF-β. It was therefore concluded that reduced responsiveness to the inhibitory effects of TGF-β may be an important event in the loss of growth control in colorectal carcinogenesis (Manning *et al.*, 1991). Whereas there is therefore good evidence to support a role for TGF-β in growth control of normal and neoplastic colonic mucosa, the expression of TGF-β in human colonic tissues is by and large poorly documented. Using a panel of antibodies which recognise different epitopes of mature TGF-β1 and pro-TGF-β1, Flanders and colleagues (1989) have demonstrated variable staining patterns in a number of tumours including carcinoma of the colon. In this study we have examined both normal and neoplastic colonic tissues for the expression of TGF-β as determined by immunohistochemistry using a polyclonal antibody raised against TGF-β1. We were particularly interested in determining whether in the normal human large bowel as in the mouse small bowel there was a differential expression of TGF-β1 along the crypt which correlated with the state of differentiation of the epithelial cells.

Correspondence: C. Paraskeva.
Received 11 November 1992; and in revised form 11 January 1993.

### Materials and methods

### *Tissues*

Formalin-fixed paraffin-embedded material obtained from the files of the Department of Pathology, Bristol Royal Infirmary were used. Forty-eight samples of colonic carcinoma and ten samples of colonic adenoma were examined. Adjacent 'normal' mucosa was included in 48 out of the total of 58 specimens examined. In addition ten samples of normal colonic mucosa taken from specimens not harbouring a tumour were also examined.

### *Immunohistochemistry*

A standard streptavidin-biotin technique was employed using a well characterised polyclonal antibody raised against TGF-β1 (Wakefield *et al.*, 1987). This antibody was raised in rabbits using synthetic peptides corresponding to amino acids 266–278 of human pro-TGF-β1 and has been shown to recognise intracellular TGF-β in tumour cells which are known to express and produce this protein (Flanders *et al.*, 1989). In our experiments trypsinisation was found to be unnecessary. In each experiment negative controls were examined where the primary antibody was omitted with or without normal rabbit serum. Slides were scored as strongly positive (+), weakly positive (±) or negative (–).

### Results

### *Mucosa*

Positive staining of epithelial cells was present in nine out of ten samples of normal colonic mucosa taken from specimens which did not harbour a tumour. Staining intensity was more marked within surface epithelial cells and became progressively weaker in cells situated more deeply within the crypts (Figure 1). In 48 out of a total of 58 tumour samples examined mucosa was also included in the tissue sections. Positive staining of mucosal epithelial cells was present in 43 out of these 48 samples (Table I). In these positive cases within areas which showed no evidence of dysplasia and no features of so-called transitional mucosa (Filipe, 1984) the staining pattern was identical to that seen in the ten samples of normal mucosa taken from specimens not harbouring a tumour. In areas situated immediately adjacent to tumour the gradient of staining was lost, with all epithelial cells showing uniform intensity of staining irrespective of whether these areas showed dysplastic changes, transitional mucosa

**Figure 1** Immunohistochemical staining for TGF-$\beta$ in colorectal mucosa.

**Table IA** Expression of TGF-$\beta$ in colonic mucosa

| | Staining intensity + | $\pm$ | – |
|---|---|---|---|
| Number of samples | 9 | 0 | 1 |
| % of total | 90 | 0 | 10 |

**Table IB** Expression of TGF-$\beta$ in colonic mucosa (taken from specimens harbouring a tumour)

| | Staining intensity + | $\pm$ | – |
|---|---|---|---|
| Number of samples | 32 | 11 | 5 |
| % of total | 67 | 23 | 10 |

changes or appeared normal. Immunoreactivity was also noted in smooth muscle cells and within some lymphoid cells in the lamina propria.

*Carcinoma*

Positive staining of tumour cells were present in 46 out of 48 samples examined (Table II, Figure 2). Immunoreactivity was largely restricted to epithelial cells and showed a diffuse cytoplasmic pattern; positive staining was also observed in occasional adipocytes, stromal fibroblasts and also within macrophages and a few lymphoid cells. Positive staining where present was seen in almost all neoplastic cells (>90%) except in three cases where a patchy distribution was

**Table II** Expression of TGF-$\beta$ in colonic carcinoma

| *Tumour grade* | Staining intensity + | $\pm$ | – |
|---|---|---|---|
| Well differentiated | 11 | 4 | 1 |
| Moderately differentiated | 12 | 3 | 1 |
| Poorly differentiated | 13 | 3 | 0 |
| Total | 36 | 10 | 2 |
| % of total | 75 | 21 | 4 |

**Figure 2** Immunohistochemical staining for TGF-$\beta$ in colorectal carcinoma.

observed with only up to 10% of the cells showing positive staining in some areas. Two of these three tumours were poorly differentiated adenocarcinomas and one was a mucinous carcinoma. Immunostaining overall did not however appear to relate to grade of differentiation (Table II).

*Adenoma*

Nine out of the ten adenomas were positive for TGF-$\beta$ (Table III). The pattern of staining was similar to that seen in invasive carcinoma in that there was diffuse cytoplasmic staining of all epithelial cells. Within the stroma positive immunoreactivity was observed in macrophages and a few lymphoid cells.

### Discussion

The expression of TGF-$\beta$ mRNA in cell lines and primary human tumours arising in different tissues is well documented

**Table III** Expression of TGF-β in colonic adenoma

| | *Staining intensity* + | ± | − |
|---|---|---|---|
| Number of samples | 8 | 1 | 1 |
| % of total | 80 | 10 | 10 |

and it appears that by and large tumour levels are enhanced compared to normal tissues (Derynck *et al.*, 1987). In this study we report the presence of TGF-β in normal large bowel mucosa, colorectal carcinomas and colorectal adenomas. Immunoreactivity for TGF-β was predominantly present in epithelial cells in the tumours examined which suggest that the site of TGF-β production is largely restricted to the epithelial component. One of the three tumours in which TGF-β showed a patchy distribution was in mucinous carcinoma which was characterised by the presence of large amounts of intercellular mucin and very little stroma. The relative lack of TGF-β expression in this tumour is in keeping with the ability of TGF-β to stimulate the formation of extracellular matrix. Although TGF-β expression is generally a reflection of cellular differentiation, tumour grade in this study was not associated with any change in TGF-β expression. In normal mucosa TGF-β immunoreactivity showed a striking distribution being present in the upper parts of the crypts which are populated by more differentiated cells, and absent deeply within the crypts in the proliferating cell compartment. This finding is similar to that observed in the rodent large bowel in a different study in which the same antibody was employed (Glick *et al.*, 1991) and conforms to the concept of TGF-β being a feature of the differentiated phenotype. This gradient of staining along the crypt was lost in both dysplastic mucosa and morphologically normal mucosa situated immediately adjacent to tumour. This loss of staining gradient in mucosa showing no significant histological abnormality compared to tumour is of potential interest. It raises the possibility that de-regulation of TGF-β expression occurs as an early event in colorectal carcinogenesis prior to adenoma formation; alternatively it could represent a field effect. To the best of our knowledge this is the first study reporting the presence and differential distribution of TGF-β in human colorectal mucosa.

**References**

BARNARD, J.A., LYONS, R.M. & MOSES, H.L. (1990). The cell biology of transforming growth factor β. *Biochim et Biophys Acta*, **1032,** 79–90.

DERYNCK, R., GOEDEL, D.V., ULRICH, A., GUTTERMAN, J.U., WILLIAMS, R.D., BRINGMAN, T.S. & BERGER, W.H. (1987). Synthesis of messenger RNAs for transforming growth factors alpha and β and the epidermal growth factor receptor by human tumours. *Cancer Res.*, **47,** 707–712.

FILIPE, M.I. (1984). Transitional mucosa. *Histopathology*, **8,** 707–708.

FLANDERS, C.K., THOMPSON, D.S., CISSEL, D.S., VAN OBBERGHEN-SCHILLING, E., BAKER, C.C., KASS, M.E., ELLINGSWORTH, L.R., ROBERTS, A.B. & SPORN, M.B. (1989). Transforming growth factor-β1: histochemical localization with antibodies to different epitopes. *J Cell Biol.*, **108,** 653–660.

GLICK, A.B., MCCUNE, B.K., ABDULKAREM, N., FLANDERS, K.C., LUMADUE, J.A., SMITH, J.M. & SPORN, M.B. (1991). Complex regulation of TGF-β expression by retinoic acid in the vitamin A-deficient rat. *Development*, **111,** 1081–1086.

MANNING, A.M., WILLIAMS, A.C., GAME, S.M. & PARASKEVA, C. (1991). Differential sensitivity of human colonic adenoma and carcinoma cells to transforming growth factor β (TGF-β): conversion of an adenoma cell line to a tumorigenic phenotype is accompanied by a reduced response to the inhibitory effects of TGF-β. *Oncogene*, **6,** 1471–1476.

ROBERTS, A.B., THOMPSON, N.L., HEINE, U., FLANDERS, C. & SPORN, M.B. (1988). Tranforming growth factor-β: possible roles in carcinogenesis. *Br. J. Cancer*, **57,** 594–600.

WAKEFIELD, L.M., SMITH, D., FLANDERS, K.C. & SPORN, M.B. (1988). Latent transforming growth factor β from human platelets. *J. Biol. Chem.*, **263,** 7646–7654.